# Insulin sensitivity in critically ill patients: are women more insulin resistant?

**DOI:** 10.1186/s13613-021-00807-7

**Published:** 2021-01-21

**Authors:** Vincent Uyttendaele, J. Geoffrey Chase, Jennifer L. Knopp, Rebecca Gottlieb, Geoffrey M. Shaw, Thomas Desaive

**Affiliations:** 1 GIGA–In silico Medicine,, University of Liège, Allée du 6 Août 19, Bât. B5a, 4000 Liège, Belgium; 2 Department of Mechanical Engineering, University of Canterbury, Private Bag 4800, Christchurch, New Zealand; 3 Medtronic Diabetes, 18000 Devonshire St, Northridge, CA 91325 USA; 4 Christchurch Hospital, Dept of Intensive Care, Christchurch, New Zealand and University of Otago, School of Medicine, Christchurch, New Zealand

**Keywords:** Insulin sensitivity, Insulin resistance, Glycaemic control, Intensive care, Metabolic variability, Hyperglycaemia, Hypoglycaemia

## Abstract

**Background:**

Glycaemic control (GC) in intensive care unit is challenging due to significant inter- and intra-patient variability, leading to increased risk of hypoglycaemia. Recent work showed higher insulin resistance in female preterm neonates. This study aims to determine if there are differences in inter- and intra-patient metabolic variability between sexes in adults, to gain in insight into any differences in metabolic response to injury. Any significant difference would suggest GC and randomised trial design should consider sex differences to personalise care.

**Methods:**

Insulin sensitivity (SI) levels and variability are identified from retrospective clinical data for men and women. Data are divided using 6-h blocks to capture metabolic evolution over time. In total, 91 male and 54 female patient GC episodes of minimum 24 h are analysed. Hypothesis testing is used to determine whether differences are significant (*P* < 0.05), and equivalence testing is used to assess whether these differences can be considered equivalent at a clinical level. Data are assessed for the raw cohort and in 100 Monte Carlo simulations analyses where the number of men and women are equal.

**Results:**

Demographic data between females and males were all similar, including GC outcomes (safety from hypoglycaemia and high (> 50%) time in target band). Females had consistently significantly lower SI levels than males, and this difference was not clinically equivalent. However, metabolic variability between sexes was never significantly different and always clinically equivalent. Thus, inter-patient variability was significantly different between males and females, but intra-patient variability was equivalent.

**Conclusion:**

Given equivalent intra-patient variability and significantly greater insulin resistance, females can receive the same benefit from safe, effective GC as males, but may require higher insulin doses to achieve the same glycaemia. Clinical trials should consider sex differences in protocol design and outcome analyses.

## Background

Stress-induced hyperglycaemia is frequent in critically ill patients, resulting from excessive glucose production and increased insulin resistance [1, 2]. The resulting abnormal increase in blood glucose (BG) levels is associated with increased morbidity and mortality [3]. Glycaemic control (GC) to lower BG to safe ranges has had beneficial impact [4–7]. However, many other studies and analyses have shown safe, effective GC is hard to achieve safely and effectively for all patients [8–16]. The increased risk of hypoglycaemia with GC and its association with increased mortality has been identified as one major safety issue for GC targeting normoglycaemic ranges [12, 17–21]. Hence, there has been a decade long debate on optimal glycaemic targets, considering possible benefits against the consequences of increased hypoglycaemic risks [22] with lower target bands [23].

A recent independent analysis simulated the Normoglycemia in Intensive Care Evaluation-Survival Using Glucose Algorithm Regulation (NICE-SUGAR) protocol with virtual patients and compared the results against clinical reported outcomes. The results suggested the outcomes of this randomised clinical trial (RCT) could be biased [24]. More specifically, this study shows poor clinical compliance to protocol may have affected the results, and thus the associated increased hypoglycaemia and risk could be a consequence of GC protocol design, rather than GC itself. Additionally, the NICE-SUGAR protocol’s lack of patient-specificity and consequent inability to safely manage inter- and intra-patient variability could also affect control performance and safety, where these factors have been widely shown to be critical for success [2, 25–32].

Patient-specific solutions using key physiological parameters to tailor control for each patient individually, including risk assessment for GC, can improve control and patient outcomes [26, 33, 34]. Such control protocols exist, and have successfully shown safe, effective control while targeting lower glycaemic ranges [35–38], without sacrificing nutrition delivery or other care aspects [39].

In a previous study, equivalence testing on insulin sensitivity (SI) levels and variability was analysed between survivors and non-survivors to understand whether these subgroups are more or less difficult to control [40]. The main outcome of this analysis showed non-survivors had higher SI levels compared to survivors, and this difference was not clinically equivalent. However, SI variability between these cohorts was clinically equivalent. These results suggest GC outcome, and thus associated mortality, is function of protocol design, rather than patient condition. Thus, high levels of safety and performance should be able to be achieved in a mixed intensive care unit (ICU) cohort, regardless of the severity of injury or eventual outcome, which is critical to seeing potential benefits [41]. These outcomes also confirm the importance for a GC design to address metabolic variability correctly, which is really what makes safe, effective GC hard to achieve [31, 40].

While quality of GC should not be influenced by patient condition, it is possible other metabolic differences could influence control if differences in patient-specific metabolic stress response existed. In particular, a previous study on neonatal ICU patients showed greater endogenous insulin secretion in girls, suggesting higher insulin resistance and a difference between sexes in this cohort [42–44]. However, to the authors’ knowledge, no analysis clearly analysed sex-related differences in the context of GC in adult ICU.

Women have been clearly under-represented in clinical trials [45, 46]. In the 1980s–1990s, the lack of women included in trials was recognised [47], despite consuming 80% of pharmaceuticals in the US at that time [48, 49]. In particular, differences in how women metabolise or clear some drugs has led to significantly different and unintended concentrations, which should necessitate different dosing instructions [50]. However, their higher metabolic variability was seen as a potential outcome bias, and, in consequence, induced a male bias in preclinical and clinical research [51].

In this study, retrospective data are used to analyse SI levels and variability between male and female adult ICU patients, to understand if there exists a difference in these subgroups. Similar to the previous study [40], a significant difference or equivalence could help understand whether GC is different and/or more difficult between males and females. Equally, given the impact of metabolic stress response on metabolism, it could also indicate whether a difference exists between the sexes in metabolic response to injury, which is currently unknown. If so, it would provide guidance on whether GC should explicitly consider sex differences in protocol and trial design, or via personalised care.

Based on previous evidence, it is hypothesised inter- and intra-patient variability between sexes are equivalent, given no bias and similar safety and performance GC outcomes. More plainly, we hypothesise insulin sensitivity levels and their hour-to-hour variability are the same given no prior evidence or indications to the contrary in adult cohorts. This study tests this hypothesis and rationale.

## Methods

### Patients and data

Retrospective clinical data from 371 patients on the Specialised Relative Insulin Nutrition Tables (SPRINT) [6] GC protocol between August 2005 and April 2007 in the Christchurch Hospital Department of Intensive Care are used. This SPRINT protocol is the precursor of the Stochastic Targeted (STAR) GC framework [52, 53], developed to modulate both nutrition and insulin. SPRINT provided safe (low incidence of moderate and severe hypoglycaemia), effective (in low mean BG and high percentage time in 4.4–8.0 mmol/L target band) control for nearly all patients, averaging 16 measurements per day [35]. SPRINT was implemented as the standard of care in a general ICU setting, and de-identified data audit and analysis were approved by the New Zealand Health and Disability Ethics Committee Upper South Regional Ethics Committee B (Ref: URB/07/15/EXP).

From this cohort, only patients who started GC within 12 h after ICU admission and received insulin for a minimum of 24 h are used to avoid any bias due to different time since ICU admission. This specification ensures a similar starting time and progression from insult toward recovery for all patients, and thus eliminates a potential source of bias or error in results.

Both cohort and per-patient data are compared. Cohort data reflect the distribution of specific outcome metrics as a whole and is highly dependent on number of hours in each patient episode, and thus in the group. In contrast, per-patient statistics reflect distribution of specific GC outcome metrics for each episode individually, by computing the median over the entire episode (thus only one median per patient or episode). Showing cohort statistics allows to determine whether GC outcome metrics are acceptable as a whole, and per-patient statistics whether they are acceptable individually for each patient.

### Patient-specific SI

Patient-specific and model-based SI is identified hourly using the clinically validated Intensive Control Insulin–Nutrition–Glucose (ICING) physiological model [54] and integral-based fitting methods [55]. SI is a time-varying, treatment independent parameter characterising patient-specific metabolic response to insulin and glucose [56]. Hence, it also reflects patient-specific general metabolic state. Consistent low SI (high insulin resistance) suggests significant stress and inflammatory state, which alleviates as the initial insult subsides [1–3, 57, 58].

While model-based SI is used to determine whether more or less insulin needs to be used to lower BG levels to a safe target range, its hour-to-hour percentage change (%ΔSI) is used to assess potential risks of metabolic variability and dysglycaemia for a given intervention over a 1–3 hourly timeframe [52, 59–61]. This variability is what makes GC difficult to achieve safely [31]. For example, at a given insulin infusion rate, a sudden increase in SI could lead to unintended hypoglycaemia. It is extremely important for a GC design to assess and manage both inter- and intra-patient variability [31]. Hence, time-varying changes in SI levels impact control difficulty and also reflects metabolic response to injury.

### Analysis

#### Raw data cohort

SI and %ΔSI are analysed using 6-h blocks between males and females over the first 72 h of control. Cumulative distribution functions are compared for each metric. Due to the large data size, hypothesis testing is performed using bootstrapping methods to examine the difference in median SI and %ΔSI between male and female cohorts [62, 63]. For each 6-h block, data are bootstrapped 1000 times with replacement to create bootstrap samples of similar size to the original cohort in that block. For each bootstrap sample, the difference in median SI and %ΔSI are calculated, and the 95% confidence interval (CI) of these differences over all 1000 runs can thus be determined. If this 95% CI does not cross zero, this difference can be considered statistically different (*P* ≤ 0.05) [62]. Because it is uncertain whether each comparison can be considered independent, a Bonferroni correction for multiple comparisons (*n* = 12) is also used for completeness [62], using the 99.6% CI to match a significance level threshold of *P* ≤ 0.004, instead of 0.05 for significance.

In the clinical environment, it is possible that a statistically significant difference (*P* < 0.05) can have minimal impact clinically and would be too small to affect decision-making. Equivalence testing is used to assess difference based on clinical significance and determine whether this difference in median SI and median %ΔSI is within a clinically set equivalence range [64]. This range was previously determined as within an absolute 12–15% difference in median values, where a typical BG measurement error cannot be detected within this range, nor will it affect a change in insulin or nutrition administration [40]. If the 95% CI (or 99.6% CI after Bonferroni correction) of percentage difference in median SI or in the absolute difference in %ΔSI is within the equivalence range, the two distributions can be considered equivalent, despite any potential statistically significant difference. Details are also available in supplemental materials/appendixes of [40], which is open access and freely available.

#### Monte Carlo simulations for robustness

While the raw data in the original cohort as presented are analysed first (91 males or 63% versus 54 females or 37%), the analysis was repeated using Monte Carlo methods to randomly create resampled sub-cohorts with the same proportion of males and females. This approach allows a fair comparison to ensure no bias results from the specific patients and proportions in the original cohort [62, 63].

New resampled male and female cohorts (*N* = 50 each) were created by randomly selecting patients from the original cohorts with replacement. In these cohorts, 8 patients (16%) were randomly selected from patients with type 2 diabetes mellitus (T2DM) so this factor was also balanced. This process was repeated 100 times, and hypothesis and equivalence testing on SI and %ΔSI were undertaken each time. The percent (%) of times the null hypothesis was rejected and equivalence accepted is calculated for each 6-h block. This secondary analysis ensures no bias due to proportions or specific patient subsets, adding robustness to the overall results. Note, this analysis does assume the patients in each group are representative of the range of behaviours, which can be further confirmed by consistency of results over the bootstrapped cases to assess any impact of outlying patients.

## Results

### Cohort selection and demographic results

**Fig. 1:**
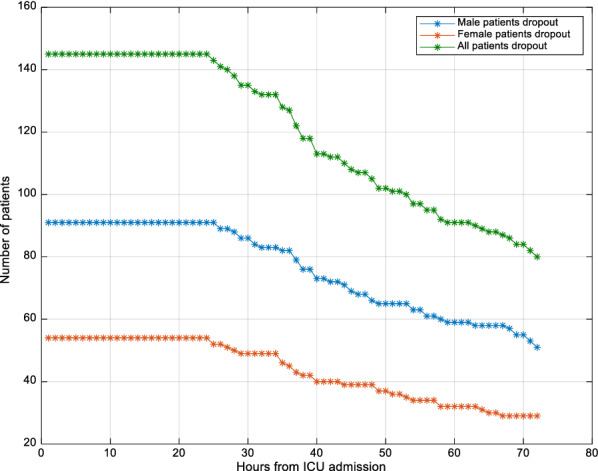
Patient dropout evolution over time

In total, 145 patients (39% of 371 patients) started GC within 12 h after ICU admission and received insulin for a minimum of 24 h. In these 145 patients, 91 (63%) were males and 54 (37%) females, which is a typical breakdown in ICU cohorts. Demographic characteristics are summarised in Table 1. In addition, patient dropout based on sex is presented in Fig. 1, where the ratio female/male patients remained in a tight range of 50–60%, which was never significantly different in proportion.

**Table 1:** Demographic summary of male and female cohorts

	Males	Females	*P*-value
Demographic statistics			
# Patients	91 (63%)	54 (37%)	
Age	67 [57, 77]	67 [58, 74]	0.63^a^
Mortality	18%	19%	1.0^b^
APACHE II score	20 [16 27]	19.5 [17, 26]	0.98^a^
First day SOFA score	6 [4 8]	5.5 [4, 8]	0.46^a^
ICU length of stay (h)	108 [67.2, 188.4]	127.2 [64.8, 213.6]	0.91^a^
SPRINT duration (h)	83 [45.5, 157.3]	86.5 [39, 167]	0.81^a^
T2DM (%)	13 (14%)	11 (20%)	0.4^b^
Per-patient GC statistics			
Median BG (mmol/L)	5.7 [5.2, 6.1]	6.0 [5.3, 6.4]	0.06^a^
Median % BG 4.4–8.0 mmol/L	83 [72, 90]	82 [67, 89]	0.3^a^
Median % BG < 4.0 mmol/L	1.4 [0, 5.5]	1.4 [0, 6.9]	0.42^a^
Median %BG < 2.2 mmol/L	0 [0, 0]	0 [0, 0]	1.0^a^
BG measurements/day	15.8 [14.4, 17.5]	15.7 [14.5, 18.2]	0.47^a^
Median insulin (U/h)	3 [2, 3]	3 [2, 3]	0.26^a^
Median feed excl. hours not fed (g/h)	3.5 [2.1, 5.5]	2.8 [1.8, 3.9]	< 0.01^a^
Median feed excl. hours not fed (%GF)	51 [30, 80]	51 [30, 75]	0.61^a^
GF (g/h)	6.5 [6.5, 7.4]	5.2 [5.2, 5.7]	< 0.01^a^
Cohort statistics			
Cohort BG (mmol/L)	5.6 [4.9, 6.6]	5.9 [5.0, 6.9]	< 0.01^ac^
% BG 4.4–8.0 mmol/L	80.6	76.9	< 0.01^b^
% BG < 4.0 mmol/L	3.2	3.8	0.11^b^
%BG < 2.2 mmol/L	0	0	1.0^b^
Insulin (U/h)	3 [1 4]	3 [2 4]	< 0.01^a^
Hours not fed	1914 (35%)	861 (27%)	< 0.01^a^
Feed excl. hours not fed (g/h)	3.8 [2.0, 5.8]	2.4 [1.7 3.9]	< 0.01^a^
Feed excl. hours not fed (%GF)	55 [29 83]	47 [29 75]	< 0.01^a^

* P*-values are not adjusted for multiple comparisons. Median [IQR] is given where appropriate. T2DM = pre-diagnosed type 2 diabetes mellitus, GF = goal feed, and BG = blood glucose. BG is hourly resampled to allow fair comparison.

Statistical difference is shown using (^a^) the Wilcoxon rank-sum test or (^b^) Fisher exact test where appropriate. (^c^) indicates clinical equivalence regardless of statistical significance, as further explained in the methods

In this analysis, the male and female cohorts were similar in all ways (Table 1). Age, diabetes, severity of injury (APACHE II and SOFA scores), length of stay, GC outcomes, measurement frequency, and insulin administration were all not significantly different. In cohort statistics, only the overall cohort BG levels, and insulin and nutrition rates were significantly different. However, the former was well within equivalence range considering measurement error and impact on outcomes [65–67], and can thus be considered not statistically different from a clinical perspective. Per-patient, only median feed administration rates achieved and goal feed were statistically different (Table 1).

Thus, the only characteristic differentiating the two cohorts at a per-patient level here was the consistently lower total grammes of dextrose administered to the female cohort. However, this difference can arise from the typically lower caloric target for women based on lower body weight [6, 68], resulting in similar grammes per kg. When nutrition was considered as the percent of the original target goal feed (GF), which is consistent and based on frame size an body weight [6, 68], nutrition was not statistically different anymore (Table 1). Overall, these two cohorts can be considered as having very similar demographic characteristics.

### Raw data cohort analysis

**Fig. 2:**
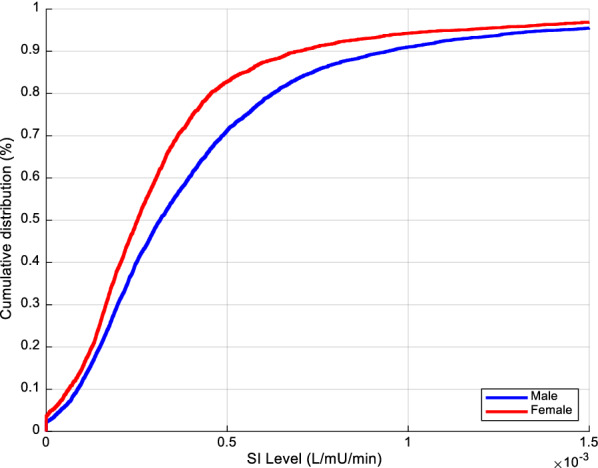
Overall cumulative SI levels (L/mU/min) between male and female cohorts

**Fig. 3:**
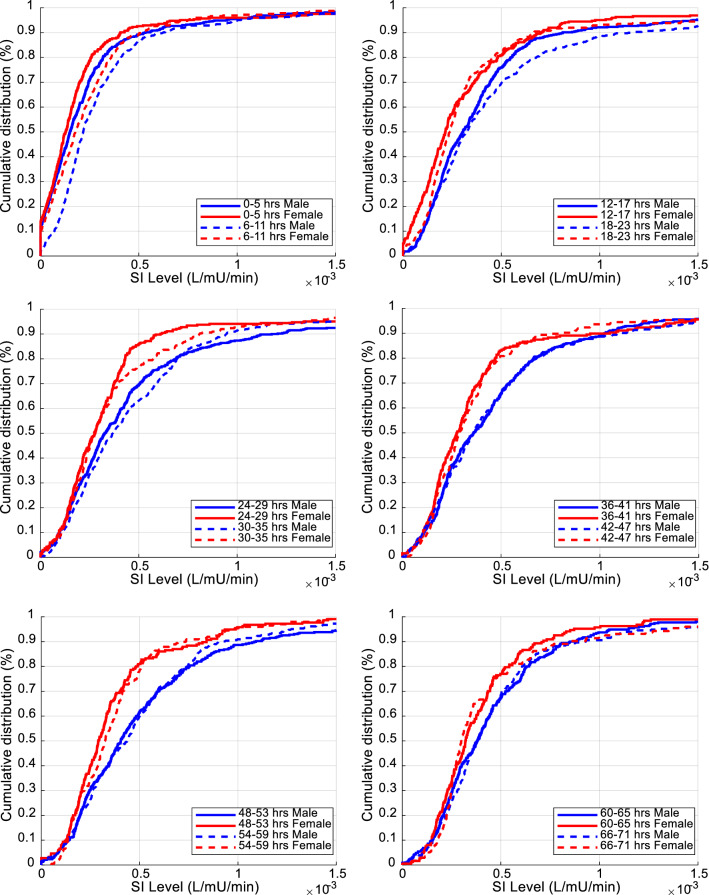
Comparison of cumulative distribution of SI levels (L/mU/min) between male and female over 6-h time intervals for the first 72 h of GC

Overall SI cumulative distribution functions for males and females are shown in Fig. 2. Clearly, the female cohort was more resistant than men (lower SI levels). SI level comparison results between males and females for every 6-h block are detailed in Table 2 and shown in Fig. 3. SI levels increased over time in both cohorts, as expected [40, 57, 58]. The 95% CI of difference in median levels between male and female never crossed zero, suggesting the difference was statistically significant not only overall, but also for each 6-h block. Considering the Bonferroni correction, 60% (7/12) of the 6-h blocks remained significantly different.

**Table 2:** Median [IQR] SI levels (L/mU/min) comparison for the first 72 h between male and female cohorts using 6-h blocks

Hours	Male cohort SI (× e−4)	female cohort SI (× e−4)	Median SI_M_−SI_F_ [95% CI] (× e−4)	
*Overall*				
0–71	3.1 [1.7 5.5]	2.5 [1.5 4.0]	*0.6 [0.5 0.8]*^*a*^	×
*Day 1*				
0–5	1.5 [0.5 2.7]	1.3 [0.5 2.3]	*0.2 [0.0 0.5]*	×
6–11	2.2 [1.3 3.7]	1.8 [0.7 3.3]	*0.4 [0.1 0.7]*	×
12–17	3.1 [1.7 4.8]	2.2 [1.1 4.2]	*0.9 [0.5 1.3]*^*a*^	×
18–23	3.3 [1.8 5.9]	2.4 [1.5 3.9]	*0.9 [0.5 1.2]*^*a*^	×
*Day 2*				
24–29	3.3 [1.8 5.7]	2.8 [1.6 4.0]	*0.5 [0.1 1.1]*	×
30–35	3.7 [2.1 6.5]	2.7 [1.8 4.6]	*1.0 [0.5 1.4]*^*a*^	×
36–41	3.6 [2.0 6.0]	2.8 [1.7 4.3]	*0.8 [0.2 1.4]*^*a*^	×
42–47	3.6 [2.0 6.0]	2.9 [1.8 4.2]	*0.7 [0.2 1.1]*^*a*^	×
*Day 3*				
48–53	4.0 [2.2 6.8]	2.9 [1.9 4.4]	*1.1 [0.6 1.6]*^*a*^	×
54–59	4.4 [2.4 6.7]	3.2 [1.9 4.8]	*1.1 [0.4 1.6]*^*a*^	×
60–65	3.8 [2.3 6.0]	3.2 [2.1 4.6]	*0.6 [0.1 1.0]*	×
66–71	3.8 [2.5 5.7]	3.0 [2.4 4.7]	*0.8 [0.4 1.2]*^*a*^	×

Equivalence is indicated by ⇔, Non-equivalence is indicated by × . Equivalence is a separate analysis to statistical difference. Hours where the medians are statistically different (95% CI does not cross zero) to *P* < 0.05 are in italic

^a^Difference remaining significant after Bonferroni correction (*P* < 0.004)

**Fig. 4:**
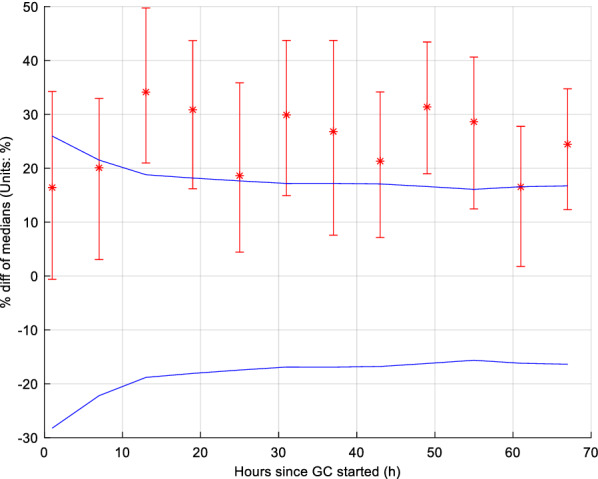
Equivalence testing on insulin sensitivity for each 6 h block. The blue lines give equivalence range for typical 9.4% BG measurement error. The 95% CI (bars) bootstrap intervals cross, or are outside, the equivalence range, indicating these 6-h blocks are not equivalent

The results of equivalence testing on SI are shown in Fig. 4. The 95% CI percentage difference in medians between males and females was always outside the clinical equivalence range. Thus, SI levels differences between male and female were statistically different, and this difference was not clinically equivalent. In particular, it showed one would expect different clinical insulin and/or nutrition administration to account for the non-equivalence.

**Fig. 5:**
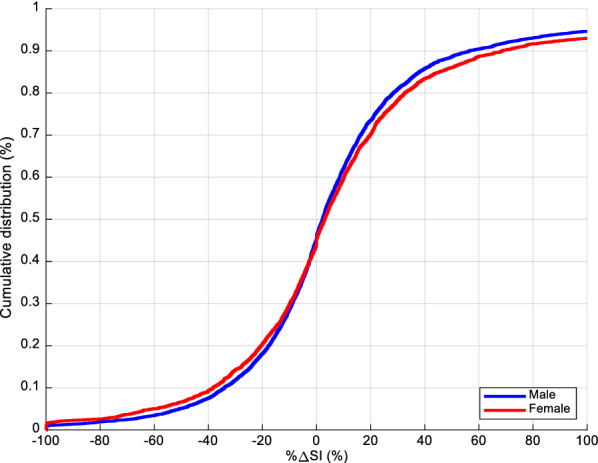
Overall cumulative %ΔSI between male and female cohorts

**Fig. 6:**
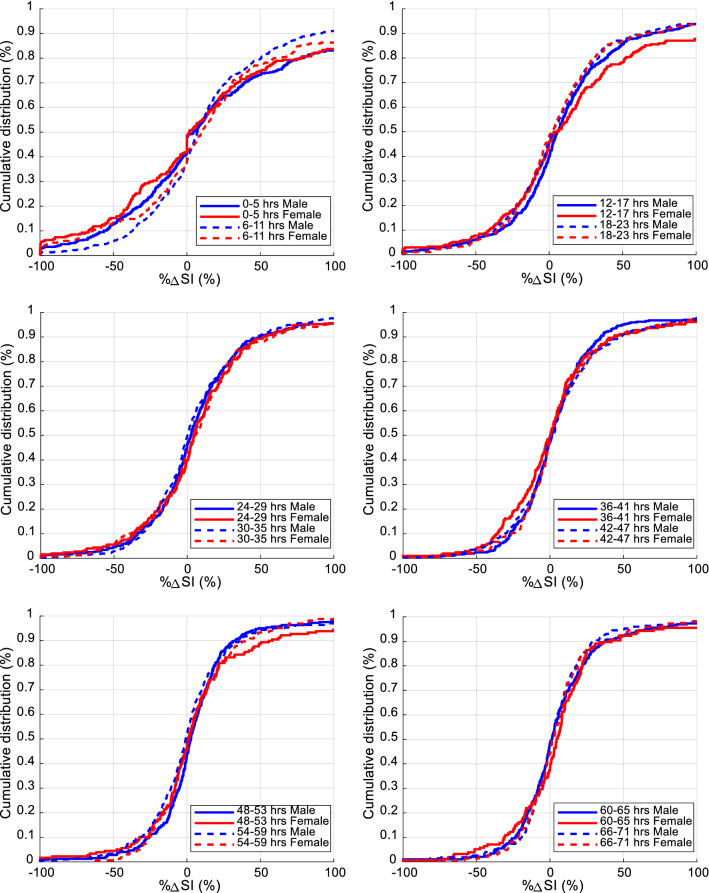
Comparison of cumulative distribution of %ΔSI (%) levels between male and female over 6-h time intervals for the first 72 h of GC

Figure 5 shows male and female cohorts overall %ΔSI. %ΔSI comparison for each 6-h block is presented in Table 3 and shown in Fig. 6. The 95% CI of bootstrapped percentage difference in median %ΔSI levels between male and female always crossed zero, except for one 6-h block (30–35 h). Male and female SI variability was thus not significantly different, especially when Bonferroni correction was considered, resulting in no 6-h blocks statistically significantly different.

**Table 3:** Median [IQR] %ΔSI (%) levels comparison between male and female cohorts using 6-h blocks

Hours	Male cohort %ΔSI	Female cohort %ΔSI	Median %ΔSIM − %ΔSIF [95% CI]	
Overall				
0–71	2.2 [− 17.8 21.6]	3.0 [− 14.4 24.9]	− 0.9 [− 2.7 1.0]	⇔
Day 1				
0–5	4.5 [− 23.1 61.3]	1.6 [− 34.5 51.1]	8.0 [− 9.7 9.8]	⇔
6–11	7.2 [− 12.7 38.7]	9.9 [− 15.4 42.0]	− 2.8 [− 9.7 4.0]	⇔
12–17	5.4 [− 10.6 27.4]	4.5 [− 16.3 37.6]	0.7 [− 8.3 7.5]	⇔
18–23	2.9 [− 15.5 24.2]	2.4 [− 14.6 25.0]	0.8 [− 4.7 7.1]	⇔
Day 2				
24–29	2.5 [− 12.5 22.1]	4.7 [− 13.0 24.9]	− 2.3 [− 6.9 1.4]	⇔
30–35	0.2 [− 15.6 23.7]	5.6 [− 12.0 24.5]	− *5.8 [*− *11.0 *− *0.7]*	⇔
36–41	1.2 [− 11.4 16.2]	0.3 [− 17.0 16.4]	1.1 [− 3.4 6.5]	⇔
42–47	2.0 [− 12.3 19.8]	0.6 [− 11.9 18.2]	1.4 [− 3.7 5.1]	⇔
Day 3				
48–53	2.7 [− 8.6 16.3]	0.7 [− 10.8 18.7]	1.6 [− 2.8 5.4]	⇔
54–59	− 0.8 [− 15.0 13.1]	1.3 [− 10.2 18.1]	− 2.3 [− 5.8 1.9]	⇔
60–65	1.3 [− 11.0 17.5]	4.5 [− 10.0 19.6]	− 3.9 [− 8.0 0.3]	⇔
66–71	1.9 [− 9.6 13.6]	1.6 [− 9.0 14.3]	− 0.6 [− 5.3 3.4]	⇔

Equivalence is indicated by ⇔, non-equivalence is indicated by × . Equivalence is a separate analysis to statistical difference. Hours where the medians are statistically different (95% CI does not cross zero) to *P* < 0.05 are in italic. No blocks were statistically significant after the Bonferroni correction (*P* < 0.004)

**Fig. 7:**
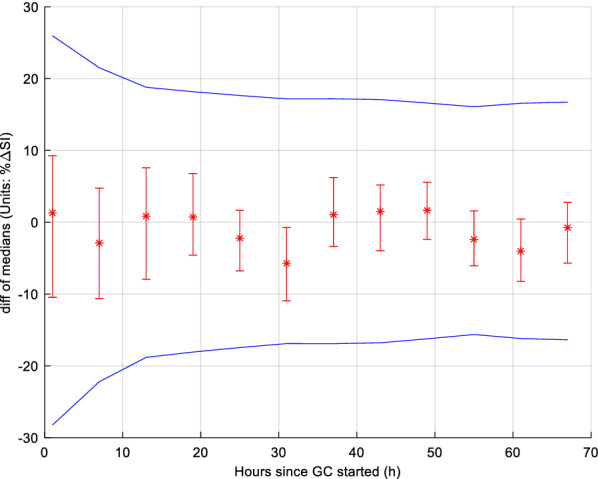
Equivalence testing on insulin sensitivity variability (%ΔSI) for each 6 h block. The blue lines give equivalence range for typical 9.4% BG measurement error. The 95% CI (bars) bootstrap intervals never cross, and are always within, the equivalence range limits, indicating these 6-hourly blocks are equivalent

Furthermore, the 95% CI difference of median %ΔSI between males and females is shown in Fig. 7 for each 6-h block in terms of equivalence. The difference was within the equivalence range for all 12 6-h blocks. Therefore, %ΔSI was not statistically significantly different for these cohorts and can also be considered equivalent.

### Monte Carlo simulations results

**Fig. 8:**
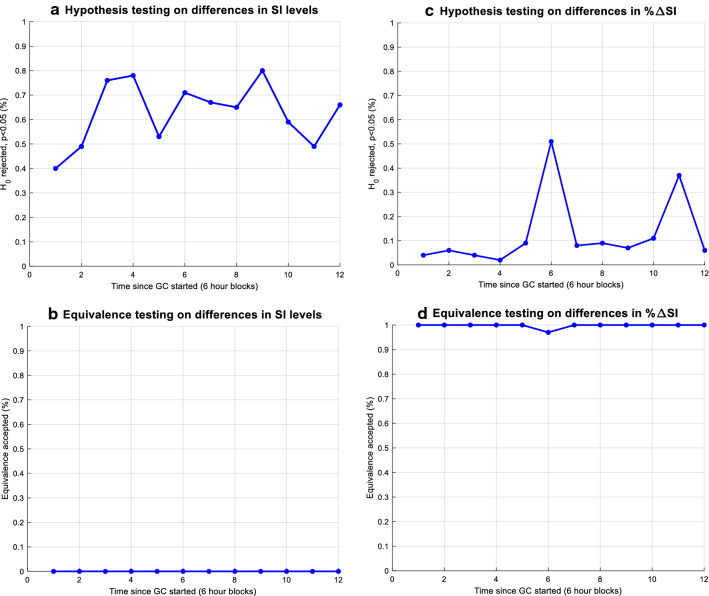
Hypothesis and equivalence testing using 6-h blocks from 100 resampled male and female (N = 50 each) sub-cohorts from which 16% have T2DM

Hypothesis and equivalence testing results for resampled (*N* = 50) male and female sub-cohorts, with the same number of T2D patients (16%) matched those of the raw, original cohort analysis. Differences in SI levels between sexes were typically significant (Fig. 8a), and never equivalent (Fig. 8b). Differences in %ΔSI were generally not significant (Fig. 8c), and almost always within equivalence range (Fig. 8d). These results confirm results from the overall population cohort analysed here.

## Discussion

### Raw cohort analysis

The results of equivalence testing on SI suggest, in addition to being statistically different, male and female median SI levels were never equivalent, clinically. In particular, it showed one would expect different clinical insulin and/or nutrition administration to account for the non-equivalence. However, the %ΔSI analysis results suggested SI variability is not statistically different and is clinically equivalent. Two observations can be taken from this set of outcomes. First, equivalent SI variability suggests both cohorts should be able to benefit from the same quality of GC, as they are equally easy/hard to control. Second, women are more insulin resistant than men (Fig. 2, Table 2). In this analysis, both cohorts received same GC quality (Table 1). All else equal, this result suggests the metabolic stress response is higher or stronger for females than for males, thus explaining this higher observed model-based insulin resistance.

These outcomes match the hypothesis of intra-patient variability being equivalent between male and female patients. However, results showed inter-patient variability in insulin sensitivity level is statistically significantly different for these groups for most blocks of time and never equivalent. Clinically, significantly different insulin dosage may be required to achieve similar glucose target in glycaemic control between males and females, but the risk associated with a specific treatment are similar between sexes.

No weight information was available for this cohort, but GF is calculated using the ACCP recommendation of 2000 kcal/day [68], and personalised for each patient according to age, sex, and body frame size using a standardised scale for consistency [39]. These three factors cover energy demands based on weight, sex, and age, where the first covers demand based on mass, the second accounts for differences in metabolic requirement per unit body weight for women, and the third accounts for decreasing demand as age rises. Personalised nutrition goals can thus vary between 1025 and 2450 kcal/day over all patients.

More specifically, as noted above, per-patient nutrition was similar in %GF delivered, but higher in grammes per hour for men due to their larger frame size. Thus, in Table 1, GF (g/h) was higher for males, as expected, reflective of their typically higher body mass. However, males and females achieved similar %GF, suggesting overall caloric goals per body mass are very similar across cohort after accounting for weight and size, given the similar age in both groups (Table 1). In addition, insulin delivery was not significantly different for both males and females.

Hence, given similar per-patient %GF delivered and total insulin administration in each group (Table 1), females received similar g/h of nutrition per body weight and demand, but were given higher insulin per body mass. More explicitly, in this comparison, %GF was normalised to mass in (large) part, but insulin delivery was not. It thus confirms females require more insulin per unit of estimated body mass to remove similar amounts of glucose given per unit of estimated body mass, supporting the lower SI found for females in this analysis.

It is important to note that while per-patient median %GF achieved was not statistically significant, by cohort, the %GF achieved considering every hour was significantly different. This difference implies the distribution of %GF received for women considering every hour is skewed slightly lower. Given similar per-patient median %GF received, women take longer to achieve essentially the same median value than men, as nutrition rates tend to rise over time in general with STAR [39, 69]. This outcome does not change the central conclusion of the research as it implies women receive more insulin and similar or less nutrition, and are thus more insulin resistant in achieving similar glycaemic outcomes.

### Monte Carlo simulations analysis

Although the proportions in this cohort reflects what is typically seen in general ICUs and are similar to those in large randomised trials [4, 8, 9], Monte Carlo simulations were used to account and reduce bias from unbalanced sexes and T2DM cohort proportions. These simulations assumed patient cohorts were representative of the general ICU population, which is the case here. Thus, resampled cohorts were created with equal numbers (*N* = 50) of males and females, and equal proportion of T2DM patients (16%), allowing fair and robust comparison between sexes.

Results for the Monte Carlo analyses matched the raw data results. The difference in SI levels can be considered significant, and this difference was never equivalent clinically. The difference in SI variability was never significant, and this difference was always clinically equivalent. Thus, these results further support and add robustness to the raw data outcome analysis of inter-patient variability being significantly different and intra-patient variability being equivalent across these subgroups of patients.

### Reasons for differences between the sexes

The SI metric used in this context comes from a validated physiological model and has been widely shown to correlate well with gold standard measures [70–72]. All else equal, the difference found in these model-based identified SI levels likely arise from two main parameters in the ICING physiological model: a higher endogenous glucose production for women than estimated; and/or a lower estimated insulin secretion rate. In the first case, higher endogenous glucose production would suggest a stronger stress response to injury, since severity are similar across the two cohorts (Apache II and SOFA scores, Table 1). In the second, the lower insulin secretion would also imply a greater suppression of insulin secretion due to stress response arising from the insult compared to men. A combination is also likely, and possible, given the impact of stress response on both issues [1–3, 73–76].

Until early 1990s, clinical trials were mainly conducted on men [47, 48]. Outcomes were thus biased, based on male clinical research results, leading to drug dosage for females being typically derived from average male requirements [49]. Women have clearly been under-represented in clinical trials [46], and are still severely under-represented today [45]. While their higher metabolic variability or difference in response to treatment was seen as a potential outcome bias [51], it has been more recently stated it should be considered as a critical factor impacting outcomes [47, 49]. More specifically, some drugs, beneficial for men, may sometimes significantly increase problems in women [77] and women can have significantly different metabolic or clearance rates for drugs resulting in very different concentrations for the same dosing protocol [50]. All these points support the importance of identifying potential sex-related effects in clinical trials and care, similar to the differences shown in this study.

In particular, many clinical trials, although including both men and women, often fail to account for potential differences in drug effectiveness or safety between men and women [50]. In the context of GC, protocols are often “one size fits all” solution, lacking the ability to account for significant inter- and intra-patient variability [33, 34], where insulin dosage is similar regardless of age, body mass, or sex. However, our result showed a clear difference between males and females for insulin requirements, due to the higher insulin resistance seen in females, which would require different dosing protocols and/or a personalised approach.

Dynamic, model-based approaches such as in STAR, or SPRINT, and their patient-specific, risk-based approach are able to capture this variability [52], and thus, intrinsically, account for differences between patients, such as sex. Such algorithms can recognise and correct the effect upon the SI resulting from any sex-related differences, where the algorithm initiation rules can be adapted based upon sex input.

Sex differences in insulin resistance, insulin secretion, glucose effectiveness or endogenous glucose production have already been shown in specific populations [43, 78–83]. The results shown in these studies sometimes contradict, but tend to say women are more sensitive to insulin than men in healthy and outpatient scenarios. In critical care patients, only one study showed a difference, demonstrating, in opposition to the above studies, higher insulin secretion and thus greater resistance in female preterm neonates compared to male preterm neonates in the neonatal ICU [43, 44]. These NICU results would not necessarily be expected to extend to adults, but the results presented show the same bias in adult ICU cohorts, suggesting a different in metabolic stress response at these two extremes of age and development.

It is important to note, many studies have analysed differences in mortality outcomes, treatment effort, or other factors between sexes in ICU patients. However, these studies often contradict. Some showed higher mortality in women [84–86], but others did not [87, 88]. The differences between sexes are thus still not completely understood in ICU, although present [89], showing the importance of assessing the related potential implications, as done in this study.

To the authors’ knowledge, there were no studies analysing endogenous glucose production or insulin secretion between sexes in adult ICU populations, which could differ in many ways due to their acute metabolic conditions. This study thus appears to be the first study suggesting women could be more resistant to insulin compared to men in this cohort, and that this outcome could be due to their potential greater response to insult induced stress.

### Limitations

A smaller cohort size of 145 patients could be a limitation. Despite the relatively small cohort size considered, an advantage of this study is the quality of the data and its detailed BG, nutrition, and insulin input information. In addition, the cohort only considers 145 patients because ensuring consistent start of GC from ICU admission of < 12 h eliminates bias due to patients being considered at different point in the evolution of stress response. Hence, the smaller cohort, while still providing sizeable data, is a result of eliminating a potential bias in this time-based analysis.

The potential impact of patient dropout on the results was not explicitly examined, but should be negligible based on prior results and the results in 6-hourly blocks showing no change over the first 72 h in overall results. In the previous comparable study by the authors [40], 80 (55%) patients had minimum 72 h of control from this same cohort of patients, and patient dropout had no effect on the results (Fig. 1). The similar ICU length of stay and GC length in Table 1 further indicate male and female patients followed similar time courses through ICU, reinforced by the similarity of SI variability being equivalent at all stages of the analysis. Finally, it should be noted, using the 6-h blocks in this analysis captures patient drop-out directly, again, as seen in the results of Figs. 3, 4, 5, 6, 7 and Tables 2, 3. Hence, patient dropout has no impact on the results presented.

The observations made rely on the identification of the SI parameter using a mathematical model, where inaccuracies could lead to bias. However, the ICING model typically performs well in the clinical ranges observed here, suggesting low inaccuracy. Furthermore, it has been validated in extensive clinical use [6, 35, 54, 90, 91].

This study does rely on retrospective data from a single-centre study, which could limit the clinical impact of these results, though, in contrast, the data reflect a generalised cohort of patients across multiple years of clinical practice. In addition, the lack of reported demographic information, such as weight, and body mass index, are a limitation to consider the caloric goals per body mass similar across cohort in this analysis, which can only be inferred at lower resolution in this study due to their use in setting goal feed rates. Finally, only sex and known diabetes mellitus have been considered in this analysis, while other confounders, such as ethnicity, could potentially influence the results. There might also be patients with unknown diabetes in the cohort, where measures of HbA1c could have helped to clearly identify these patients, but are not available here.

Based on the presented results, future work should further explore potential known and existing sex-related physiological differences, such as body composition, as it would be theoretically useful to assess any potential impact on decision-making and further explain these observations. In addition, other metrics inputs such as sex hormones, should be studied as they may have exerted effect upon GC outcome differences. Overall, this information could be useful to further improve and tailor treatment to patient-specific needs.

## Conclusions

This study compared identified SI and %ΔSI across male and female cohorts using hypothesis and equivalence testing. SI was shown statistically significantly lower for females and this difference is clinically not equivalent to males. However, %ΔSI between males and females was not statistically different, and clinically equivalent. These results strongly suggest women in a general ICU cohort may have stronger metabolic stress response than men, but this latter outcome remains to be confirmed clinically. These results also suggest higher insulin requirements for females, while equal safety and efficacy should be able to be achieved for both cohorts, as reflected in the equivalent variability. Future GC RCTs should thus also consider randomising and analysing male–female subgroups for differences in primary and secondary outcomes.

## Data Availability

The de-identified data sets used and/or analysed during the current study are available from the corresponding author on reasonable request. However, a subset of the data are publicly available in another journal: Chase J.G. et al. A benchmark data set for model-based glycemic control in critical care, J Diabetes Sci Technol 2008, 2(4): 584–94.

## Abbreviations

BG: Blood glucose
CI: Confidence interval
GC: Glycaemic control
GF: Goal feed
ICING: Intensive control insulin–nutrition–glucose
ICU: Intensive care unit
IQR: Interquartile range
NICE-SUGAR: Normoglycaemia in intensive care evaluation-survival using glucose algorithm regulation
RCT: Randomised clinical trial
SI: Insulin sensitivity
STAR: Stochastic Targeted
T2DM: Type 2 diabetes mellitus
%ΔSI: Hour-to-hour percentage change in SI
